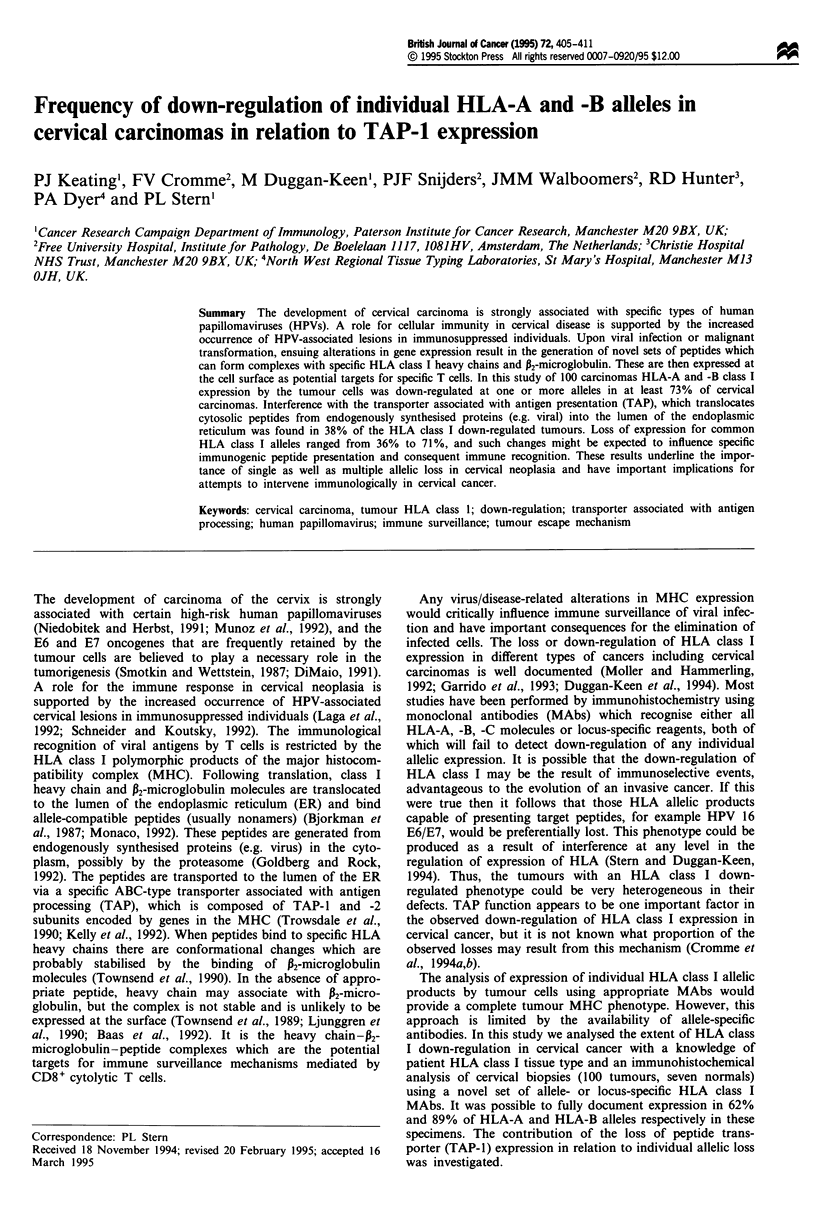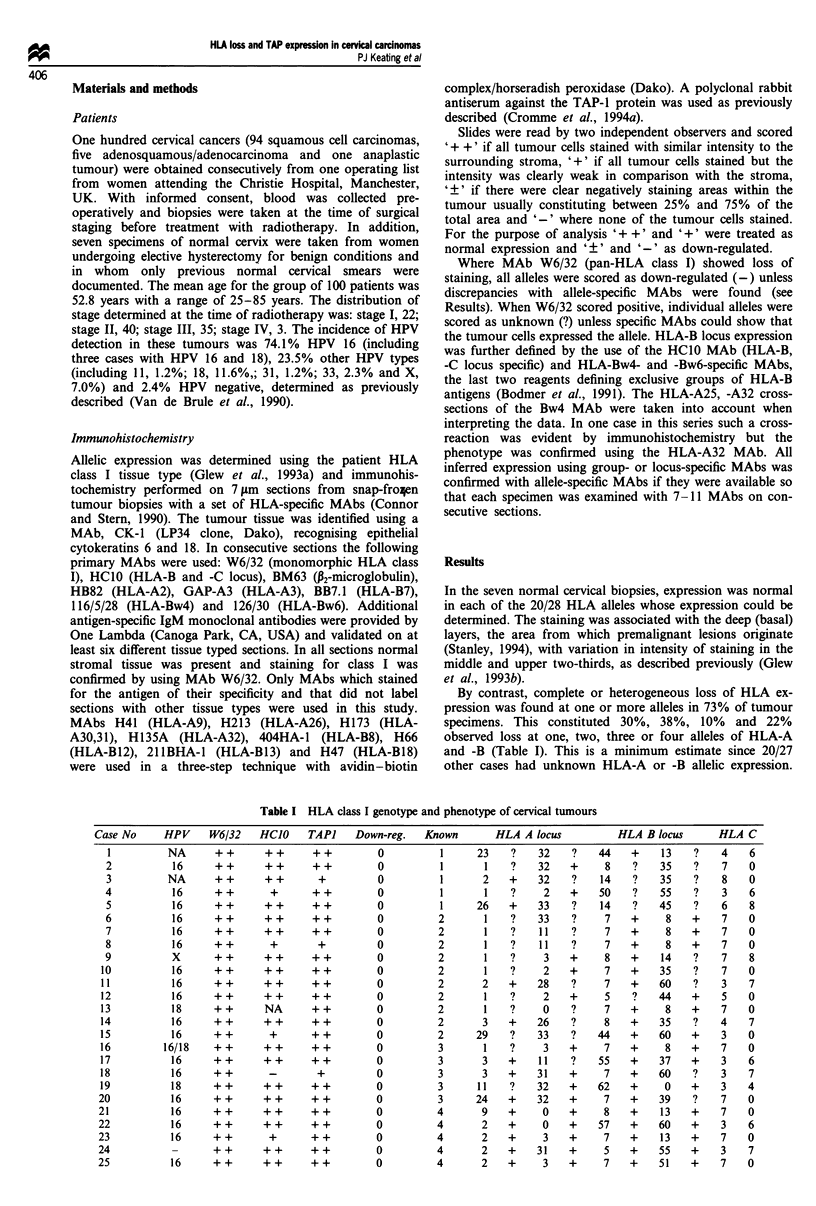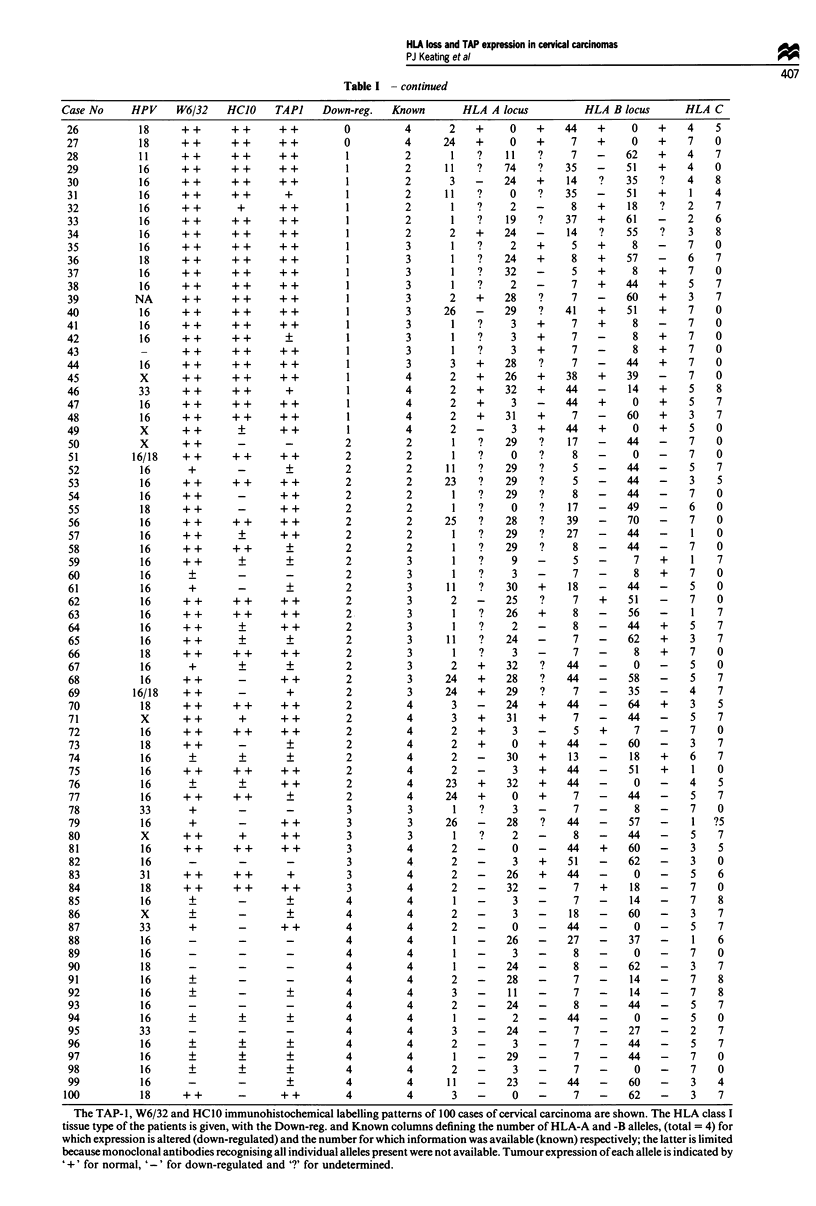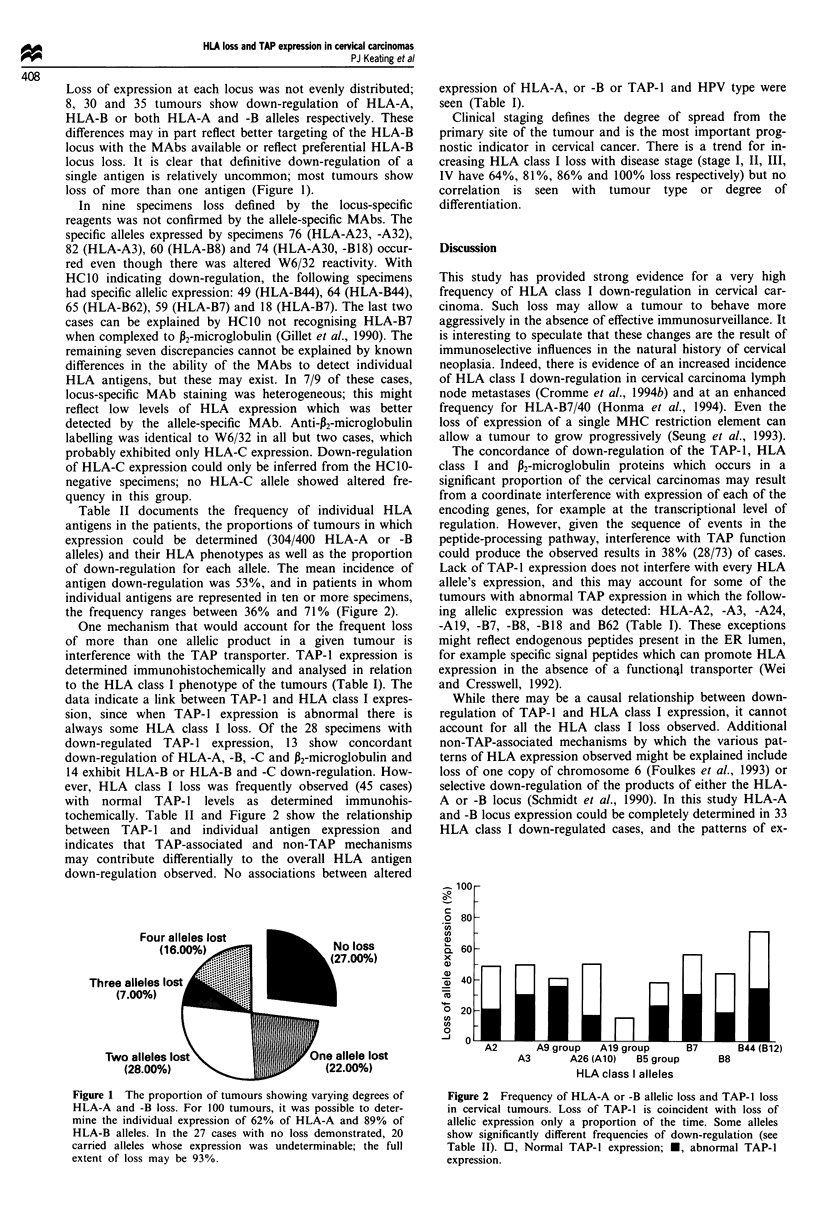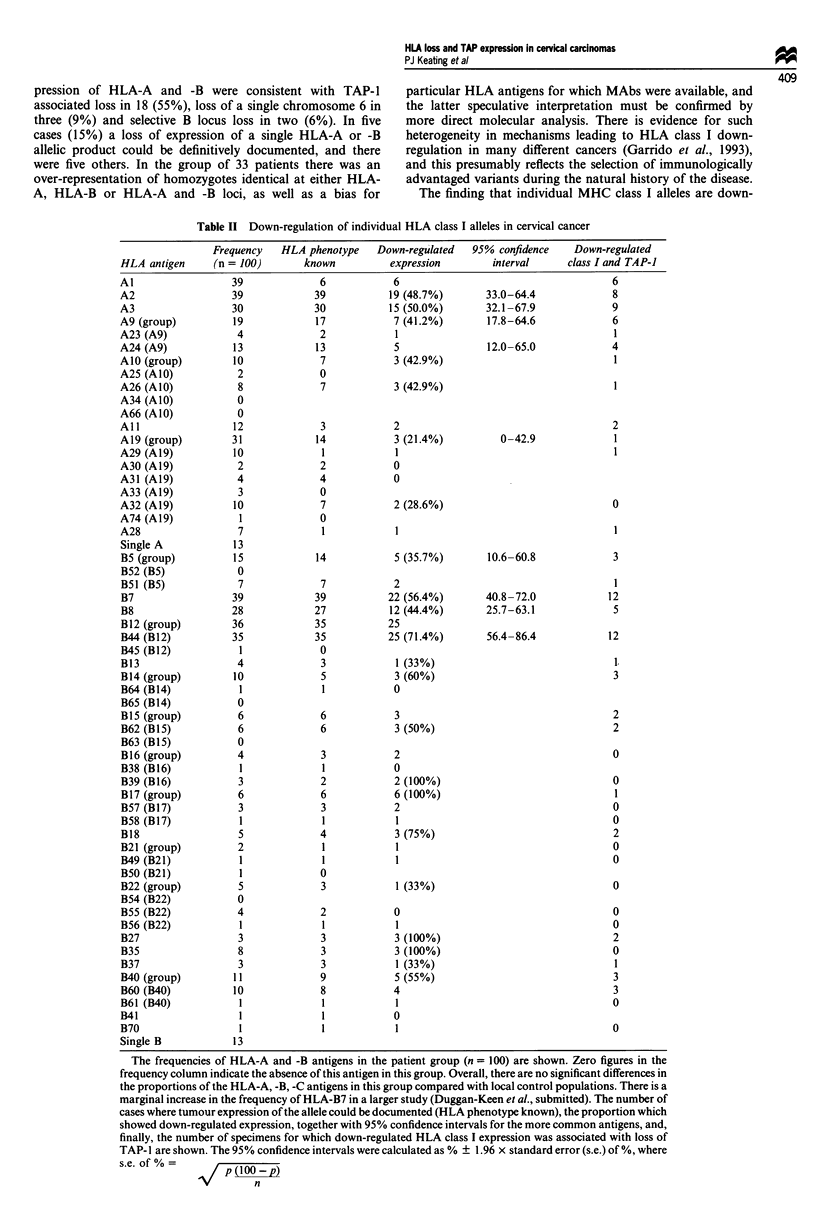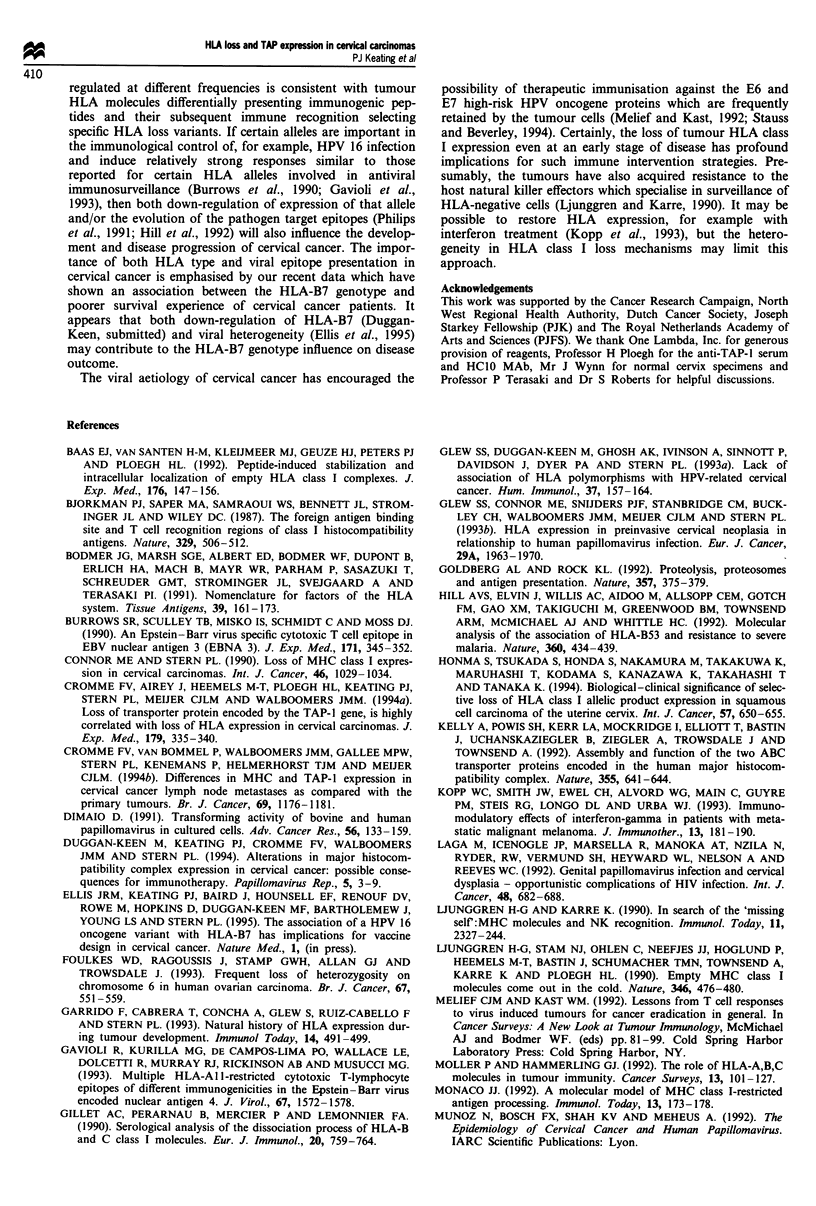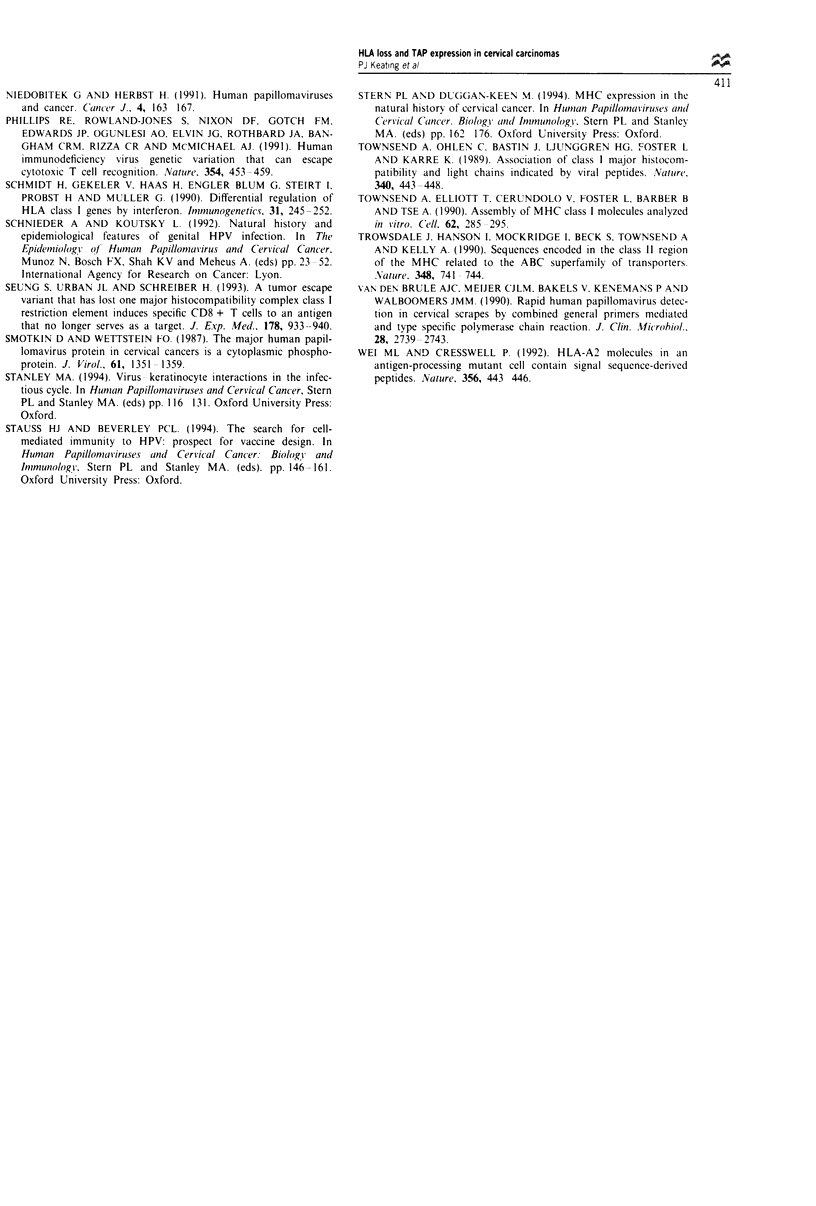# Frequency of down-regulation of individual HLA-A and -B alleles in cervical carcinomas in relation to TAP-1 expression.

**DOI:** 10.1038/bjc.1995.346

**Published:** 1995-08

**Authors:** P. J. Keating, F. V. Cromme, M. Duggan-Keen, P. J. Snijders, J. M. Walboomers, R. D. Hunter, P. A. Dyer, P. L. Stern

**Affiliations:** Cancer Research Campaign Department of Immunology, Paterson Institute for Cancer Research, Manchester, UK.

## Abstract

The development of cervical carcinoma is strongly associated with specific types of human papillomaviruses (HPVs). A role for cellular immunity in cervical disease is supported by the increased occurrence of HPV-associated lesions in immunosuppressed individuals. Upon viral infection or malignant transformation, ensuing alterations in gene expression result in the generation of novel sets of peptides which can form complexes with specific HLA class I heavy chains and beta 2-microglobulin. These are then expressed at the cell surface as potential targets for specific T cells. In this study of 100 carcinomas HLA-A and -B class I expression by the tumour cells was down-regulated at one or more alleles in at least 73% of cervical carcinomas. Interference with the transporter associated with antigen presentation (TAP), which translocates cytosolic peptides from endogenously synthesised proteins (e.g. viral) into the lumen of the endoplasmic reticulum was found in 38% of the HLA class I down-regulated tumours. Loss of expression for common HLA class I alleles ranged from 36% to 71%, and such changes might be expected to influence specific immunogenic peptide presentation and consequent immune recognition. These results underline the importance of single as well as multiple allelic loss in cervical neoplasia and have important implications for attempts to intervene immunologically in cervical cancer.


British Journal of Cancer (1995) 72, 405-411
© 1995 Stockton Press All rights reserved 0007-0920/95 $12.00

### Frequency of down-regulation of individual HLA-A and -B alleles in cervical carcinomas in relation to TAP-1 expression

PJ Keating[1], FV Cromme[2], M Duggan-Keen[1], PJF Snijders[2], JMM Walboomers[2], RD Hunter[3], PA Dyer[4] and PL Stern[1]

[1]*Cancer Research Campaign Department of Immunology, Paterson Institute for Cancer Research, Manchester M20 9BX, UK;* [2]*Free University Hospital, Institute for Pathology, De Boelelaan 1117, 1081HV, Amsterdam, The Netherlands;* [3]*Christie Hospital NHS Trust, Manchester M20 9BX, UK;* [4]*North West Regional Tissue Typing Laboratories, St Mary's Hospital, Manchester M13 0JH, UK.*

**Summary** The development of cervical carcinoma is strongly associated with specific types of human papillomaviruses (HPVs). A role for cellular immunity in cervical disease is supported by the increased occurrence of HPV-associated lesions in immunosuppressed individuals. Upon viral infection or malignant transformation, ensuing alterations in gene expression result in the generation of novel sets of peptides which can form complexes with specific HLA class I heavy chains and $\beta_2$-microglobulin. These are then expressed at the cell surface as potential targets for specific T cells. In this study of 100 carcinomas HLA-A and -B class I expression by the tumour cells was down-regulated at one or more alleles in at least 73% of cervical carcinomas. Interference with the transporter associated with antigen presentation (TAP), which translocates cytosolic peptides from endogenously synthesised proteins (e.g. viral) into the lumen of the endoplasmic reticulum was found in 38% of the HLA class I down-regulated tumours. Loss of expression for common HLA class I alleles ranged from 36% to 71%, and such changes might be expected to influence specific immunogenic peptide presentation and consequent immune recognition. These results underline the importance of single as well as multiple allelic loss in cervical neoplasia and have important implications for attempts to intervene immunologically in cervical cancer.

**Keywords**: cervical carcinoma, tumour HLA class 1; down-regulation; transporter associated with antigen processing; human papillomavirus; immune surveillance; tumour escape mechanism

The development of carcinoma of the cervix is strongly associated with certain high-risk human papillomaviruses (Niedobitek and Herbst, 1991; Munoz *et al.*, 1992), and the E6 and E7 oncogenes that are frequently retained by the tumour cells are believed to play a necessary role in the tumorigenesis (Smotkin and Wettstein, 1987; DiMaio, 1991). A role for the immune response in cervical neoplasia is supported by the increased occurrence of HPV-associated cervical lesions in immunosuppressed individuals (Laga *et al.*, 1992; Schneider and Koutsky, 1992). The immunological recognition of viral antigens by T cells is restricted by the HLA class I polymorphic products of the major histocompatibility complex (MHC). Following translation, class I heavy chain and $\beta_2$-microglobulin molecules are translocated to the lumen of the endoplasmic reticulum (ER) and bind allele-compatible peptides (usually nonamers) (Bjorkman *et al.*, 1987; Monaco, 1992). These peptides are generated from endogenously synthesised proteins (e.g. virus) in the cytoplasm, possibly by the proteasome (Goldberg and Rock, 1992). The peptides are transported to the lumen of the ER via a specific ABC-type transporter associated with antigen processing (TAP), which is composed of TAP-1 and -2 subunits encoded by genes in the MHC (Trowsdale *et al.*, 1990; Kelly *et al.*, 1992). When peptides bind to specific HLA heavy chains there are conformational changes which are probably stabilised by the binding of $\beta_2$-microglobulin molecules (Townsend *et al.*, 1990). In the absence of appropriate peptide, heavy chain may associate with $\beta_2$-microglobulin, but the complex is not stable and is unlikely to be expressed at the surface (Townsend *et al.*, 1989; Ljunggren *et al.*, 1990; Baas *et al.*, 1992). It is the heavy chain–$\beta_2$-microglobulin–peptide complexes which are the potential targets for immune surveillance mechanisms mediated by $CD8^+$ cytolytic T cells.

Correspondence: PL Stern
Received 18 November 1994; revised 20 February 1995; accepted 16 March 1995

Any virus/disease-related alterations in MHC expression would critically influence immune surveillance of viral infection and have important consequences for the elimination of infected cells. The loss or down-regulation of HLA class I expression in different types of cancers including cervical carcinomas is well documented (Moller and Hammerling, 1992; Garrido *et al.*, 1993; Duggan-Keen *et al.*, 1994). Most studies have been performed by immunohistochemistry using monoclonal antibodies (MAbs) which recognise either all HLA-A, -B, -C molecules or locus-specific reagents, both of which will fail to detect down-regulation of any individual allelic expression. It is possible that the down-regulation of HLA class I may be the result of immunoselective events, advantageous to the evolution of an invasive cancer. If this were true then it follows that those HLA allelic products capable of presenting target peptides, for example HPV 16 E6/E7, would be preferentially lost. This phenotype could be produced as a result of interference at any level in the regulation of expression of HLA (Stern and Duggan-Keen, 1994). Thus, the tumours with an HLA class I down-regulated phenotype could be very heterogeneous in their defects. TAP function appears to be one important factor in the observed down-regulation of HLA class I expression in cervical cancer, but it is not known what proportion of the observed losses may result from this mechanism (Cromme *et al.*, 1994*a*,*b*).

The analysis of expression of individual HLA class I allelic products by tumour cells using appropriate MAbs would provide a complete tumour MHC phenotype. However, this approach is limited by the availability of allele-specific antibodies. In this study we analysed the extent of HLA class I down-regulation in cervical cancer with a knowledge of patient HLA class I tissue type and an immunohistochemical analysis of cervical biopsies (100 tumours, seven normals) using a novel set of allele- or locus-specific HLA class I MAbs. It was possible to fully document expression in 62% and 89% of HLA-A and HLA-B alleles respectively in these specimens. The contribution of the loss of peptide transporter (TAP-1) expression in relation to individual allelic loss was investigated.

### Materials and methods

### *Patients*

One hundred cervical cancers (94 squamous cell carcinomas, five adenosquamous/adenocarcinoma and one anaplastic tumour) were obtained consecutively from one operating list from women attending the Christie Hospital, Manchester, UK. With informed consent, blood was collected pre-operatively and biopsies were taken at the time of surgical staging before treatment with radiotherapy. In addition, seven specimens of normal cervix were taken from women undergoing elective hysterectomy for benign conditions and in whom only previous normal cervical smears were documented. The mean age for the group of 100 patients was 52.8 years with a range of 25–85 years. The distribution of stage determined at the time of radiotherapy was: stage I, 22; stage II, 40; stage III, 35; stage IV, 3. The incidence of HPV detection in these tumours was 74.1% HPV 16 (including three cases with HPV 16 and 18), 23.5% other HPV types (including 11, 1.2%; 18, 11.6%,; 31, 1.2%; 33, 2.3% and X, 7.0%) and 2.4% HPV negative, determined as previously described (Van de Brule *et al.*, 1990).

### *Immunohistochemistry*

Allelic expression was determined using the patient HLA class I tissue type (Glew *et al.*, 1993a) and immunohistochemistry performed on 7 μm sections from snap-frozen tumour biopsies with a set of HLA-specific MAbs (Connor and Stern, 1990). The tumour tissue was identified using a MAb, CK-1 (LP34 clone, Dako), recognising epithelial cytokeratins 6 and 18. In consecutive sections the following primary MAbs were used: W6/32 (monomorphic HLA class I), HC10 (HLA-B and -C locus), BM63 ($\beta_2$-microglobulin), HB82 (HLA-A2), GAP-A3 (HLA-A3), BB7.1 (HLA-B7), 116/5/28 (HLA-Bw4) and 126/30 (HLA-Bw6). Additional antigen-specific IgM monoclonal antibodies were provided by One Lambda (Canoga Park, CA, USA) and validated on at least six different tissue typed sections. In all sections normal stromal tissue was present and staining for class I was confirmed by using MAb W6/32. Only MAbs which stained for the antigen of their specificity and that did not label sections with other tissue types were used in this study. MAbs H41 (HLA-A9), H213 (HLA-A26), H173 (HLA-A30,31), H135A (HLA-A32), 404HA-1 (HLA-B8), H66 (HLA-B12), 211BHA-1 (HLA-B13) and H47 (HLA-B18) were used in a three-step technique with avidin–biotin complex/horseradish peroxidase (Dako). A polyclonal rabbit antiserum against the TAP-1 protein was used as previously described (Cromme *et al.*, 1994*a*).

Slides were read by two independent observers and scored '+ +' if all tumour cells stained with similar intensity to the surrounding stroma, '+' if all tumour cells stained but the intensity was clearly weak in comparison with the stroma, '±' if there were clear negatively staining areas within the tumour usually constituting between 25% and 75% of the total area and '−' where none of the tumour cells stained. For the purpose of analysis '+ +' and '+' were treated as normal expression and '±' and '−' as down-regulated.

Where MAb W6/32 (pan-HLA class I) showed loss of staining, all alleles were scored as down-regulated (−) unless discrepancies with allele-specific MAbs were found (see Results). When W6/32 scored positive, individual alleles were scored as unknown (?) unless specific MAbs could show that the tumour cells expressed the allele. HLA-B locus expression was further defined by the use of the HC10 MAb (HLA-B, -C locus specific) and HLA-Bw4- and -Bw6-specific MAbs, the last two reagents defining exclusive groups of HLA-B antigens (Bodmer *et al.*, 1991). The HLA-A25, -A32 cross-sections of the Bw4 MAb were taken into account when interpreting the data. In one case in this series such a cross-reaction was evident by immunohistochemistry but the phenotype was confirmed using the HLA-A32 MAb. All inferred expression using group- or locus-specific MAbs was confirmed with allele-specific MAbs if they were available so that each specimen was examined with 7–11 MAbs on consecutive sections.

### Results

In the seven normal cervical biopsies, expression was normal in each of the 20/28 HLA alleles whose expression could be determined. The staining was associated with the deep (basal) layers, the area from which premalignant lesions originate (Stanley, 1994), with variation in intensity of staining in the middle and upper two-thirds, as described previously (Glew *et al.*, 1993*b*).

By contrast, complete or heterogeneous loss of HLA expression was found at one or more alleles in 73% of tumour specimens. This constituted 30%, 38%, 10% and 22% observed loss at one, two, three or four alleles of HLA-A and -B (Table I). This is a minimum estimate since 20/27 other cases had unknown HLA-A or -B allelic expression.

**Table I** HLA class I genotype and phenotype of cervical tumours

| *Case No* | *HPV* | *W6/32* | *HC10* | *TAP1* | *Down-reg.* | *Known* | *HLA A locus* | | | | *HLA B locus* | | | | *HLA C* | |
|---|---|---|---|---|---|---|---|---|---|---|---|---|---|---|---|---|
| 1 | NA | + + | + + | + + | 0 | 1 | 23 | ? | 32 | ? | 44 | + | 13 | ? | 4 | 6 |
| 2 | 16 | + + | + + | + + | 0 | 1 | 1 | ? | 32 | + | 8 | ? | 35 | ? | 7 | 0 |
| 3 | NA | + + | + + | + | 0 | 1 | 2 | + | 32 | ? | 14 | ? | 35 | ? | 8 | 0 |
| 4 | 16 | + + | + | + + | 0 | 1 | 1 | ? | 2 | + | 50 | ? | 55 | ? | 3 | 6 |
| 5 | 16 | + + | + + | + + | 0 | 1 | 26 | + | 33 | ? | 14 | ? | 45 | ? | 6 | 8 |
| 6 | 16 | + + | + + | + + | 0 | 2 | 1 | ? | 33 | ? | 7 | + | 8 | + | 7 | 0 |
| 7 | 16 | + + | + + | + + | 0 | 2 | 1 | ? | 11 | ? | 7 | + | 8 | + | 7 | 0 |
| 8 | 16 | + + | + | + | 0 | 2 | 1 | ? | 11 | ? | 7 | + | 8 | + | 7 | 0 |
| 9 | X | + + | + + | + + | 0 | 2 | 1 | ? | 3 | + | 8 | + | 14 | ? | 7 | 8 |
| 10 | 16 | + + | + + | + + | 0 | 2 | 1 | ? | 2 | + | 7 | + | 35 | ? | 7 | 0 |
| 11 | 16 | + + | + + | + + | 0 | 2 | 2 | + | 28 | ? | 7 | + | 60 | ? | 3 | 7 |
| 12 | 16 | + + | + + | + + | 0 | 2 | 1 | ? | 2 | + | 5 | ? | 44 | + | 5 | 0 |
| 13 | 18 | + + | NA | + + | 0 | 2 | 1 | ? | 0 | ? | 7 | + | 8 | + | 7 | 0 |
| 14 | 16 | + + | + + | + + | 0 | 2 | 3 | + | 26 | ? | 8 | + | 35 | ? | 4 | 7 |
| 15 | 16 | + + | + | + + | 0 | 2 | 29 | ? | 33 | ? | 44 | + | 60 | + | 3 | 0 |
| 16 | 16/18 | + + | + + | + + | 0 | 3 | 1 | ? | 3 | + | 7 | + | 8 | + | 7 | 0 |
| 17 | 16 | + + | + + | + + | 0 | 3 | 3 | + | 11 | ? | 55 | + | 37 | + | 3 | 6 |
| 18 | 16 | + + | − | + | 0 | 3 | 3 | + | 31 | + | 7 | + | 60 | ? | 3 | 7 |
| 19 | 18 | + + | + + | + + | 0 | 3 | 11 | ? | 32 | + | 62 | + | 0 | + | 3 | 4 |
| 20 | 16 | + + | + + | + + | 0 | 3 | 24 | + | 32 | + | 7 | + | 39 | ? | 7 | 0 |
| 21 | 16 | + + | + + | + + | 0 | 4 | 9 | + | 0 | + | 8 | + | 13 | + | 7 | 0 |
| 22 | 16 | + + | + + | + + | 0 | 4 | 2 | + | 0 | + | 57 | + | 60 | + | 3 | 6 |
| 23 | 16 | + + | + | + + | 0 | 4 | 2 | + | 3 | + | 7 | + | 13 | + | 7 | 0 |
| 24 | − | + + | + + | + + | 0 | 4 | 2 | + | 31 | + | 5 | + | 55 | + | 3 | 7 |
| 25 | 16 | + + | + + | + + | 0 | 4 | 2 | + | 3 | + | 7 | + | 51 | + | 7 | 0 |

**Table I** – *continued*

| *Case No* | *HPV* | *W6/32* | *HC10* | *TAP1* | *Down-reg.* | *Known* | *HLA A locus* | | | | *HLA B locus* | | | | *HLA C* | |
|---|---|---|---|---|---|---|---|---|---|---|---|---|---|---|---|---|
| 26 | 18 | ++ | ++ | ++ | 0 | 4 | 2 | + | 0 | + | 44 | + | 0 | + | 4 | 5 |
| 27 | 18 | ++ | ++ | ++ | 0 | 4 | 24 | + | 0 | + | 7 | + | 0 | + | 7 | 0 |
| 28 | 11 | ++ | ++ | ++ | 1 | 2 | 1 | ? | 11 | ? | 7 | – | 62 | + | 4 | 7 |
| 29 | 16 | ++ | ++ | ++ | 1 | 2 | 11 | ? | 74 | ? | 35 | – | 51 | + | 4 | 0 |
| 30 | 16 | ++ | ++ | ++ | 1 | 2 | 3 | – | 24 | + | 14 | ? | 35 | ? | 4 | 8 |
| 31 | 16 | ++ | ++ | + | 1 | 2 | 11 | ? | 0 | ? | 35 | – | 51 | + | 1 | 4 |
| 32 | 16 | ++ | + | ++ | 1 | 2 | 1 | ? | 2 | – | 8 | + | 18 | ? | 2 | 7 |
| 33 | 16 | ++ | ++ | ++ | 1 | 2 | 1 | ? | 19 | ? | 37 | + | 61 | – | 2 | 6 |
| 34 | 16 | ++ | ++ | ++ | 1 | 2 | 2 | + | 24 | – | 14 | ? | 55 | ? | 3 | 8 |
| 35 | 16 | ++ | ++ | ++ | 1 | 3 | 1 | ? | 2 | + | 5 | + | 8 | – | 7 | 0 |
| 36 | 18 | ++ | ++ | ++ | 1 | 3 | 1 | ? | 24 | + | 8 | + | 57 | – | 6 | 7 |
| 37 | 16 | ++ | ++ | ++ | 1 | 3 | 1 | ? | 32 | – | 5 | + | 8 | + | 7 | 0 |
| 38 | 16 | ++ | ++ | ++ | 1 | 3 | 1 | ? | 2 | – | 7 | + | 44 | + | 5 | 7 |
| 39 | NA | ++ | ++ | ++ | 1 | 3 | 2 | + | 28 | ? | 7 | – | 60 | + | 3 | 7 |
| 40 | 16 | ++ | ++ | ++ | 1 | 3 | 26 | – | 29 | ? | 41 | + | 51 | + | 7 | 0 |
| 41 | 16 | ++ | ++ | ++ | 1 | 3 | 1 | ? | 3 | + | 7 | + | 8 | – | 7 | 0 |
| 42 | 16 | ++ | ++ | ± | 1 | 3 | 1 | ? | 3 | + | 7 | – | 8 | + | 7 | 0 |
| 43 | – | ++ | ++ | ++ | 1 | 3 | 1 | ? | 3 | + | 7 | – | 8 | + | 7 | 0 |
| 44 | 16 | ++ | ++ | ++ | 1 | 3 | 3 | + | 28 | ? | 7 | – | 44 | + | 7 | 0 |
| 45 | X | ++ | ++ | ++ | 1 | 4 | 2 | + | 26 | + | 38 | + | 39 | – | 7 | 0 |
| 46 | 33 | ++ | ++ | + | 1 | 4 | 2 | + | 32 | + | 44 | – | 14 | + | 5 | 8 |
| 47 | 16 | ++ | ++ | ++ | 1 | 4 | 2 | + | 3 | – | 44 | + | 0 | + | 5 | 7 |
| 48 | 16 | ++ | ++ | ++ | 1 | 4 | 2 | + | 31 | + | 7 | – | 60 | + | 3 | 7 |
| 49 | X | ++ | ± | ++ | 1 | 4 | 2 | – | 3 | + | 44 | + | 0 | + | 5 | 0 |
| 50 | X | ++ | – | – | 2 | 2 | 1 | ? | 29 | ? | 17 | – | 44 | – | 7 | 0 |
| 51 | 16/18 | ++ | ++ | ++ | 2 | 2 | 1 | ? | 0 | ? | 8 | – | 0 | – | 7 | 0 |
| 52 | 16 | + | – | ± | 2 | 2 | 11 | ? | 29 | ? | 5 | – | 44 | – | 5 | 7 |
| 53 | 16 | ++ | ++ | ++ | 2 | 2 | 23 | ? | 29 | ? | 5 | – | 44 | – | 3 | 5 |
| 54 | 16 | ++ | – | ++ | 2 | 2 | 1 | ? | 29 | ? | 8 | – | 44 | – | 7 | 0 |
| 55 | 18 | ++ | – | ++ | 2 | 2 | 1 | ? | 0 | ? | 17 | – | 49 | – | 6 | 0 |
| 56 | 16 | ++ | ++ | ++ | 2 | 2 | 25 | ? | 28 | ? | 39 | – | 70 | – | 7 | 0 |
| 57 | 16 | ++ | ± | ++ | 2 | 2 | 1 | ? | 29 | ? | 27 | – | 44 | – | 1 | 0 |
| 58 | 16 | ++ | ++ | ± | 2 | 2 | 1 | ? | 29 | ? | 8 | – | 44 | – | 7 | 0 |
| 59 | 16 | ++ | ± | ± | 2 | 3 | 1 | ? | 9 | – | 5 | – | 7 | + | 1 | 7 |
| 60 | 16 | ± | – | – | 2 | 3 | 1 | ? | 3 | – | 7 | – | 8 | + | 7 | 0 |
| 61 | 16 | + | – | ± | 2 | 3 | 11 | ? | 30 | + | 18 | – | 44 | – | 5 | 0 |
| 62 | 16 | ++ | ++ | ++ | 2 | 3 | 2 | – | 25 | ? | 7 | + | 51 | – | 7 | 0 |
| 63 | 16 | ++ | ++ | ++ | 2 | 3 | 1 | ? | 26 | + | 8 | – | 56 | – | 1 | 7 |
| 64 | 16 | ++ | ± | ++ | 2 | 3 | 1 | ? | 2 | – | 8 | – | 44 | + | 5 | 7 |
| 65 | 16 | ++ | ± | ± | 2 | 3 | 11 | ? | 24 | – | 7 | – | 62 | + | 3 | 7 |
| 66 | 18 | ++ | ++ | ++ | 2 | 3 | 1 | ? | 3 | – | 7 | – | 8 | + | 7 | 0 |
| 67 | 16 | + | ± | ± | 2 | 3 | 2 | + | 32 | ? | 44 | – | 0 | – | 5 | 0 |
| 68 | 16 | ++ | – | ++ | 2 | 3 | 24 | + | 28 | ? | 44 | – | 58 | – | 5 | 7 |
| 69 | 16/18 | ++ | – | + | 2 | 3 | 24 | + | 29 | ? | 7 | – | 35 | – | 4 | 7 |
| 70 | 18 | ++ | ++ | ++ | 2 | 4 | 3 | – | 24 | + | 44 | – | 64 | + | 3 | 5 |
| 71 | X | ++ | + | ++ | 2 | 4 | 3 | + | 31 | + | 7 | – | 44 | – | 5 | 7 |
| 72 | 16 | ++ | ++ | ++ | 2 | 4 | 2 | + | 3 | – | 5 | + | 7 | – | 7 | 0 |
| 73 | 18 | ++ | – | ± | 2 | 4 | 2 | + | 0 | + | 44 | – | 60 | – | 3 | 7 |
| 74 | 16 | ± | ± | ± | 2 | 4 | 2 | – | 30 | + | 13 | – | 18 | + | 6 | 7 |
| 75 | 16 | ++ | ++ | ++ | 2 | 4 | 2 | – | 3 | + | 44 | – | 51 | + | 1 | 0 |
| 76 | 16 | ± | ± | ++ | 2 | 4 | 23 | + | 32 | + | 44 | – | 0 | – | 4 | 5 |
| 77 | 16 | ++ | ++ | ± | 2 | 4 | 24 | + | 0 | + | 7 | – | 44 | – | 5 | 7 |
| 78 | 33 | + | – | – | 3 | 3 | 1 | ? | 3 | – | 7 | – | 8 | – | 7 | 0 |
| 79 | 16 | + | – | ++ | 3 | 3 | 26 | – | 28 | ? | 44 | – | 57 | – | 1 | ?5 |
| 80 | X | ++ | + | ++ | 3 | 3 | 1 | ? | 2 | – | 8 | – | 44 | – | 5 | 7 |
| 81 | 16 | ++ | ++ | ++ | 3 | 4 | 2 | – | 0 | – | 44 | + | 60 | – | 3 | 5 |
| 82 | 16 | – | – | – | 3 | 4 | 2 | – | 3 | + | 51 | – | 62 | – | 3 | 0 |
| 83 | 31 | ++ | ++ | + | 3 | 4 | 2 | – | 26 | + | 44 | – | 0 | – | 5 | 6 |
| 84 | 18 | ++ | ++ | ++ | 3 | 4 | 2 | – | 32 | – | 7 | + | 18 | – | 7 | 0 |
| 85 | 16 | ± | – | ± | 4 | 4 | 1 | – | 3 | – | 7 | – | 14 | – | 7 | 8 |
| 86 | X | ± | – | ± | 4 | 4 | 2 | – | 3 | – | 18 | – | 60 | – | 3 | 7 |
| 87 | 33 | + | – | ++ | 4 | 4 | 2 | – | 0 | – | 44 | – | 0 | – | 5 | 7 |
| 88 | 16 | – | – | – | 4 | 4 | 1 | – | 26 | – | 27 | – | 37 | – | 1 | 6 |
| 89 | 16 | – | – | – | 4 | 4 | 1 | – | 3 | – | 8 | – | 0 | – | 7 | 0 |
| 90 | 18 | – | – | – | 4 | 4 | 1 | – | 24 | – | 8 | – | 62 | – | 3 | 7 |
| 91 | 16 | ± | – | – | 4 | 4 | 2 | – | 28 | – | 7 | – | 14 | – | 7 | 8 |
| 92 | 16 | ± | – | ± | 4 | 4 | 3 | – | 11 | – | 7 | – | 14 | – | 7 | 8 |
| 93 | 16 | – | – | – | 4 | 4 | 2 | – | 24 | – | 8 | – | 44 | – | 5 | 7 |
| 94 | 16 | ± | ± | ± | 4 | 4 | 1 | – | 2 | – | 44 | – | 0 | – | 5 | 0 |
| 95 | 33 | – | – | – | 4 | 4 | 3 | – | 24 | – | 7 | – | 27 | – | 2 | 7 |
| 96 | 16 | ± | ± | ± | 4 | 4 | 2 | – | 3 | – | 7 | – | 44 | – | 5 | 7 |
| 97 | 16 | ± | ± | ± | 4 | 4 | 1 | – | 29 | – | 7 | – | 44 | – | 7 | 0 |
| 98 | 16 | ± | ± | ± | 4 | 4 | 2 | – | 3 | – | 7 | – | 0 | – | 7 | 0 |
| 99 | 16 | – | – | ± | 4 | 4 | 11 | – | 23 | – | 44 | – | 60 | – | 3 | 4 |
| 100 | 18 | ++ | – | ++ | 4 | 4 | 3 | – | 0 | – | 7 | – | 62 | – | 3 | 7 |

The TAP-1, W6/32 and HC10 immunohistochemical labelling patterns of 100 cases of cervical carcinoma are shown. The HLA class I tissue type of the patients is given, with the Down-reg. and Known columns defining the number of HLA-A and -B alleles, (total = 4) for which expression is altered (down-regulated) and the number for which information was available (known) respectively; the latter is limited because monoclonal antibodies recognising all individual alleles present were not available. Tumour expression of each allele is indicated by '+' for normal, '–' for down-regulated and '?' for undetermined.

Loss of expression at each locus was not evenly distributed; 8, 30 and 35 tumours show down-regulation of HLA-A, HLA-B or both HLA-A and -B alleles respectively. These differences may in part reflect better targeting of the HLA-B locus with the MAbs available or reflect preferential HLA-B locus loss. It is clear that definitive down-regulation of a single antigen is relatively uncommon; most tumours show loss of more than one antigen (Figure 1).

In nine specimens loss defined by the locus-specific reagents was not confirmed by the allele-specific MAbs. The specific alleles expressed by specimens 76 (HLA-A23, -A32), 82 (HLA-A3), 60 (HLA-B8) and 74 (HLA-A30, -B18) occurred even though there was altered W6/32 reactivity. With HC10 indicating down-regulation, the following specimens had specific allelic expression: 49 (HLA-B44), 64 (HLA-B44), 65 (HLA-B62), 59 (HLA-B7) and 18 (HLA-B7). The last two cases can be explained by HC10 not recognising HLA-B7 when complexed to $\beta_2$-microglobulin (Gillet *et al.*, 1990). The remaining seven discrepancies cannot be explained by known differences in the ability of the MAbs to detect individual HLA antigens, but these may exist. In 7/9 of these cases, locus-specific MAb staining was heterogeneous; this might reflect low levels of HLA expression which was better detected by the allele-specific MAb. Anti-$\beta_2$-microglobulin labelling was identical to W6/32 in all but two cases, which probably exhibited only HLA-C expression. Down-regulation of HLA-C expression could only be inferred from the HC10-negative specimens; no HLA-C allele showed altered frequency in this group.

Table II documents the frequency of individual HLA antigens in the patients, the proportions of tumours in which expression could be determined (304/400 HLA-A or -B alleles) and their HLA phenotypes as well as the proportion of down-regulation for each allele. The mean incidence of antigen down-regulation was 53%, and in patients in whom individual antigens are represented in ten or more specimens, the frequency ranges between 36% and 71% (Figure 2).

One mechanism that would account for the frequent loss of more than one allelic product in a given tumour is interference with the TAP transporter. TAP-1 expression is determined immunohistochemically and analysed in relation to the HLA class I phenotype of the tumours (Table I). The data indicate a link between TAP-1 and HLA class I expression, since when TAP-1 expression is abnormal there is always some HLA class I loss. Of the 28 specimens with down-regulated TAP-1 expression, 13 show concordant down-regulation of HLA-A, -B, -C and $\beta_2$-microglobulin and 14 exhibit HLA-B or HLA-B and -C down-regulation. However, HLA class I loss was frequently observed (45 cases) with normal TAP-1 levels as determined immunohistochemically. Table II and Figure 2 show the relationship between TAP-1 and individual antigen expression and indicates that TAP-associated and non-TAP mechanisms may contribute differentially to the overall HLA antigen down-regulation observed. No associations between altered expression of HLA-A, or -B or TAP-1 and HPV type were seen (Table I).

Clinical staging defines the degree of spread from the primary site of the tumour and is the most important prognostic indicator in cervical cancer. There is a trend for increasing HLA class I loss with disease stage (stage I, II, III, IV have 64%, 81%, 86% and 100% loss respectively) but no correlation is seen with tumour type or degree of differentiation.

### Discussion

This study has provided strong evidence for a very high frequency of HLA class I down-regulation in cervical carcinoma. Such loss may allow a tumour to behave more aggressively in the absence of effective immunosurveillance. It is interesting to speculate that these changes are the result of immunoselective influences in the natural history of cervical neoplasia. Indeed, there is evidence of an increased incidence of HLA class I down-regulation in cervical carcinoma lymph node metastases (Cromme *et al.*, 1994*b*) and at an enhanced frequency for HLA-B7/40 (Honma *et al.*, 1994). Even the loss of expression of a single MHC restriction element can allow a tumour to grow progressively (Seung *et al.*, 1993).

The concordance of down-regulation of the TAP-1, HLA class I and $\beta_2$-microglobulin proteins which occurs in a significant proportion of the cervical carcinomas may result from a coordinate interference with expression of each of the encoding genes, for example at the transcriptional level of regulation. However, given the sequence of events in the peptide-processing pathway, interference with TAP function could produce the observed results in 38% (28/73) of cases. Lack of TAP-1 expression does not interfere with every HLA allele's expression, and this may account for some of the tumours with abnormal TAP expression in which the following allelic expression was detected: HLA-A2, -A3, -A24, -A19, -B7, -B8, -B18 and B62 (Table I). These exceptions might reflect endogenous peptides present in the ER lumen, for example specific signal peptides which can promote HLA expression in the absence of a functional transporter (Wei and Cresswell, 1992).

While there may be a causal relationship between down-regulation of TAP-1 and HLA class I expression, it cannot account for all the HLA class I loss observed. Additional non-TAP-associated mechanisms by which the various patterns of HLA expression observed might be explained include loss of one copy of chromosome 6 (Foulkes *et al.*, 1993) or selective down-regulation of the products of either the HLA-A or -B locus (Schmidt *et al.*, 1990). In this study HLA-A and -B locus expression could be completely determined in 33 HLA class I down-regulated cases, and the patterns of ex-

**Figure 1** The proportion of tumours showing varying degrees of HLA-A and -B loss. For 100 tumours, it was possible to determine the individual expression of 62% of HLA-A and 89% of HLA-B alleles. In the 27 cases with no loss demonstrated, 20 carried alleles whose expression was undeterminable; the full extent of loss may be 93%.

**Figure 2** Frequency of HLA-A or -B allelic loss and TAP-1 loss in cervical tumours. Loss of TAP-1 is coincident with loss of allelic expression only a proportion of the time. Some alleles show significantly different frequencies of down-regulation (see Table II). □, Normal TAP-1 expression; ■, abnormal TAP-1 expression.

pression of HLA-A and -B were consistent with TAP-1 associated loss in 18 (55%), loss of a single chromosome 6 in three (9%) and selective B locus loss in two (6%). In five cases (15%) a loss of expression of a single HLA-A or -B allelic product could be definitively documented, and there were five others. In the group of 33 patients there was an over-representation of homozygotes identical at either HLA-A, HLA-B or HLA-A and -B loci, as well as a bias for particular HLA antigens for which MAbs were available, and the latter speculative interpretation must be confirmed by more direct molecular analysis. There is evidence for such heterogeneity in mechanisms leading to HLA class I down-regulation in many different cancers (Garrido *et al.*, 1993), and this presumably reflects the selection of immunologically advantaged variants during the natural history of the disease.

The finding that individual MHC class I alleles are down-

**Table II** Down-regulation of individual HLA class I alleles in cervical cancer

| *HLA antigen* | *Frequency (n = 100)* | *HLA phenotype known* | *Down-regulated expression* | *95% confidence interval* | *Down-regulated class I and TAP-1* |
|---|---|---|---|---|---|
| A1 | 39 | 6 | 6 | | 6 |
| A2 | 39 | 39 | 19 (48.7%) | 33.0–64.4 | 8 |
| A3 | 30 | 30 | 15 (50.0%) | 32.1–67.9 | 9 |
| A9 (group) | 19 | 17 | 7 (41.2%) | 17.8–64.6 | 6 |
| A23 (A9) | 4 | 2 | 1 | | 1 |
| A24 (A9) | 13 | 13 | 5 | 12.0–65.0 | 4 |
| A10 (group) | 10 | 7 | 3 (42.9%) | | 1 |
| A25 (A10) | 2 | 0 | | | |
| A26 (A10) | 8 | 7 | 3 (42.9%) | | 1 |
| A34 (A10) | 0 | | | | |
| A66 (A10) | 0 | | | | |
| A11 | 12 | 3 | 2 | | 2 |
| A19 (group) | 31 | 14 | 3 (21.4%) | 0–42.9 | 1 |
| A29 (A19) | 10 | 1 | 1 | | 1 |
| A30 (A19) | 2 | 2 | 0 | | |
| A31 (A19) | 4 | 4 | 0 | | |
| A33 (A19) | 3 | 0 | | | |
| A32 (A19) | 10 | 7 | 2 (28.6%) | | 0 |
| A74 (A19) | 1 | 0 | | | |
| A28 | 7 | 1 | 1 | | 1 |
| Single A | 13 | | | | |
| B5 (group) | 15 | 14 | 5 (35.7%) | 10.6–60.8 | 3 |
| B52 (B5) | 0 | | | | |
| B51 (B5) | 7 | 7 | 2 | | 1 |
| B7 | 39 | 39 | 22 (56.4%) | 40.8–72.0 | 12 |
| B8 | 28 | 27 | 12 (44.4%) | 25.7–63.1 | 5 |
| B12 (group) | 36 | 35 | 25 | | |
| B44 (B12) | 35 | 35 | 25 (71.4%) | 56.4–86.4 | 12 |
| B45 (B12) | 1 | 0 | | | |
| B13 | 4 | 3 | 1 (33%) | | 1 |
| B14 (group) | 10 | 5 | 3 (60%) | | 3 |
| B64 (B14) | 1 | 1 | 0 | | |
| B65 (B14) | 0 | | | | |
| B15 (group) | 6 | 6 | 3 | | 2 |
| B62 (B15) | 6 | 6 | 3 (50%) | | 2 |
| B63 (B15) | 0 | | | | |
| B16 (group) | 4 | 3 | 2 | | 0 |
| B38 (B16) | 1 | 1 | 0 | | |
| B39 (B16) | 3 | 2 | 2 (100%) | | 0 |
| B17 (group) | 6 | 6 | 6 (100%) | | 1 |
| B57 (B17) | 3 | 3 | 2 | | 0 |
| B58 (B17) | 1 | 1 | 1 | | 0 |
| B18 | 5 | 4 | 3 (75%) | | 2 |
| B21 (group) | 2 | 1 | 1 | | 0 |
| B49 (B21) | 1 | 1 | 1 | | 0 |
| B50 (B21) | 1 | 0 | | | |
| B22 (group) | 5 | 3 | 1 (33%) | | 0 |
| B54 (B22) | 0 | | | | |
| B55 (B22) | 4 | 2 | 0 | | 0 |
| B56 (B22) | 1 | 1 | 1 | | 0 |
| B27 | 3 | 3 | 3 (100%) | | 2 |
| B35 | 8 | 3 | 3 (100%) | | 0 |
| B37 | 3 | 3 | 1 (33%) | | 1 |
| B40 (group) | 11 | 9 | 5 (55%) | | 3 |
| B60 (B40) | 10 | 8 | 4 | | 3 |
| B61 (B40) | 1 | 1 | 1 | | 0 |
| B41 | 1 | 1 | 0 | | |
| B70 | 1 | 1 | 1 | | 0 |
| Single B | 13 | | | | |

The frequencies of HLA-A and -B antigens in the patient group ($n = 100$) are shown. Zero figures in the frequency column indicate the absence of this antigen in this group. Overall, there are no significant differences in the proportions of the HLA-A, -B, -C antigens in this group compared with local control populations. There is a marginal increase in the frequency of HLA-B7 in a larger study (Duggan-Keen *et al.*, submitted). The number of cases where tumour expression of the allele could be documented (HLA phenotype known), the proportion which showed down-regulated expression, together with 95% confidence intervals for the more common antigens, and, finally, the number of specimens for which down-regulated HLA class I expression was associated with loss of TAP-1 are shown. The 95% confidence intervals were calculated as % ± 1.96 × standard error (s.e.) of %, where s.e. of % = $\sqrt{\frac{p\,(100-p)}{n}}$

regulated at different frequencies is consistent with tumour HLA molecules differentially presenting immunogenic peptides and their subsequent immune recognition selecting specific HLA loss variants. If certain alleles are important in the immunological control of, for example, HPV 16 infection and induce relatively strong responses similar to those reported for certain HLA alleles involved in antiviral immunosurveillance (Burrows *et al.*, 1990; Gavioli *et al.*, 1993), then both down-regulation of expression of that allele and/or the evolution of the pathogen target epitopes (Philips *et al.*, 1991; Hill *et al.*, 1992) will also influence the development and disease progression of cervical cancer. The importance of both HLA type and viral epitope presentation in cervical cancer is emphasised by our recent data which have shown an association between the HLA-B7 genotype and poorer survival experience of cervical cancer patients. It appears that both down-regulation of HLA-B7 (Duggan-Keen, submitted) and viral heterogeneity (Ellis *et al.*, 1995) may contribute to the HLA-B7 genotype influence on disease outcome.

The viral aetiology of cervical cancer has encouraged the possibility of therapeutic immunisation against the E6 and E7 high-risk HPV oncogene proteins which are frequently retained by the tumour cells (Melief and Kast, 1992; Stauss and Beverley, 1994). Certainly, the loss of tumour HLA class I expression even at an early stage of disease has profound implications for such immune intervention strategies. Presumably, the tumours have also acquired resistance to the host natural killer effectors which specialise in surveillance of HLA-negative cells (Ljunggren and Karre, 1990). It may be possible to restore HLA expression, for example with interferon treatment (Kopp *et al.*, 1993), but the heterogeneity in HLA class I loss mechanisms may limit this approach.

**Acknowledgements**
This work was supported by the Cancer Research Campaign, North West Regional Health Authority, Dutch Cancer Society, Joseph Starkey Fellowship (PJK) and The Royal Netherlands Academy of Arts and Sciences (PJFS). We thank One Lambda, Inc. for generous provision of reagents, Professor H Ploegh for the anti-TAP-1 serum and HC10 MAb, Mr J Wynn for normal cervix specimens and Professor P Terasaki and Dr S Roberts for helpful discussions.

### References

BAAS EJ, VAN SANTEN H-M, KLEIJMEER MJ, GEUZE HJ, PETERS PJ AND PLOEGH HL. (1992). Peptide-induced stabilization and intracellular localization of empty HLA class I complexes. *J. Exp. Med.*, **176**, 147–156.

BJORKMAN PJ, SAPER MA, SAMRAOUI WS, BENNETT JL, STROMINGER JL AND WILEY DC. (1987). The foreign antigen binding site and T cell recognition regions of class I histocompatibility antigens. *Nature*, **329**, 506–512.

BODMER JG, MARSH SGE, ALBERT ED, BODMER WF, DUPONT B, ERLICH HA, MACH B, MAYR WR, PARHAM P, SASAZUKI T, SCHREUDER GMT, STROMINGER JL, SVEJGAARD A AND TERASAKI PI. (1991). Nomenclature for factors of the HLA system. *Tissue Antigens*, **39**, 161–173.

BURROWS SR, SCULLEY TB, MISKO IS, SCHMIDT C AND MOSS DJ. (1990). An Epstein–Barr virus specific cytotoxic T cell epitope in EBV nuclear antigen 3 (EBNA 3). *J. Exp. Med.*, **171**, 345–352.

CONNOR ME AND STERN PL. (1990). Loss of MHC class I expression in cervical carcinomas. *Int. J. Cancer*, **46**, 1029–1034.

CROMME FV, AIREY J, HEEMELS M-T, PLOEGH HL, KEATING PJ, STERN PL, MEIJER CJLM AND WALBOOMERS JMM. (1994*a*). Loss of transporter protein encoded by the TAP-1 gene, is highly correlated with loss of HLA expression in cervical carcinomas. *J. Exp. Med.*, **179**, 335–340.

CROMME FV, VAN BOMMEL P, WALBOOMERS JMM, GALLEE MPW, STERN PL, KENEMANS P, HELMERHORST TJM AND MEIJER CJLM. (1994*b*). Differences in MHC and TAP-1 expression in cervical cancer lymph node metastases as compared with the primary tumours. *Br. J. Cancer*, **69**, 1176–1181.

DIMAIO D. (1991). Transforming activity of bovine and human papillomavirus in cultured cells. *Adv. Cancer Res.*, **56**, 133–159.

DUGGAN-KEEN M, KEATING PJ, CROMME FV, WALBOOMERS JMM AND STERN PL. (1994). Alterations in major histocompatibility complex expression in cervical cancer: possible consequences for immunotherapy. *Papillomavirus Rep.*, **5**, 3–9.

ELLIS JRM, KEATING PJ, BAIRD J, HOUNSELL EF, RENOUF DV, ROWE M, HOPKINS D, DUGGAN-KEEN MF, BARTHOLEMEW J, YOUNG LS AND STERN PL. (1995). The association of a HPV 16 oncogene variant with HLA-B7 has implications for vaccine design in cervical cancer. *Nature Med.*, **1**, (in press).

FOULKES WD, RAGOUSSIS J, STAMP GWH, ALLAN GJ AND TROWSDALE J. (1993). Frequent loss of heterozygosity on chromosome 6 in human ovarian carcinoma. *Br. J. Cancer*, **67**, 551–559.

GARRIDO F, CABRERA T, CONCHA A, GLEW S, RUIZ-CABELLO F AND STERN PL. (1993). Natural history of HLA expression during tumour development. *Immunol Today*, **14**, 491–499.

GAVIOLI R, KURILLA MG, DE CAMPOS-LIMA PO, WALLACE LE, DOLCETTI R, MURRAY RJ, RICKINSON AB AND MUSUCCI MG. (1993). Multiple HLA-A11-restricted cytotoxic T-lymphocyte epitopes of different immunogenicities in the Epstein–Barr virus encoded nuclear antigen 4. *J. Virol.*, **67**, 1572–1578.

GILLET AC, PERARNAU B, MERCIER P AND LEMONNIER FA. (1990). Serological analysis of the dissociation process of HLA-B and C class I molecules. *Eur. J. Immunol.*, **20**, 759–764.

GLEW SS, DUGGAN-KEEN M, GHOSH AK, IVINSON A, SINNOTT P, DAVIDSON J, DYER PA AND STERN PL. (1993*a*). Lack of association of HLA polymorphisms with HPV-related cervical cancer. *Hum. Immunol.*, **37**, 157–164.

GLEW SS, CONNOR ME, SNIJDERS PJF, STANBRIDGE CM, BUCKLEY CH, WALBOOMERS JMM, MEIJER CJLM AND STERN PL. (1993*b*). HLA expression in preinvasive cervical neoplasia in relationship to human papillomavirus infection. *Eur. J. Cancer*, **29A**, 1963–1970.

GOLDBERG AL AND ROCK KL. (1992). Proteolysis, proteosomes and antigen presentation. *Nature*, **357**, 375–379.

HILL AVS, ELVIN J, WILLIS AC, AIDOO M, ALLSOPP CEM, GOTCH FM, GAO XM, TAKIGUCHI M, GREENWOOD BM, TOWNSEND ARM, MCMICHAEL AJ AND WHITTLE HC. (1992). Molecular analysis of the association of HLA-B53 and resistance to severe malaria. *Nature*, **360**, 434–439.

HONMA S, TSUKADA S, HONDA S, NAKAMURA M, TAKAKUWA K, MARUHASHI T, KODAMA S, KANAZAWA K, TAKAHASHI T AND TANAKA K. (1994). Biological–clinical significance of selective loss of HLA class I allelic product expression in squamous cell carcinoma of the uterine cervix. *Int. J. Cancer*, **57**, 650–655.

KELLY A, POWIS SH, KERR LA, MOCKRIDGE I, ELLIOTT T, BASTIN J, UCHANSKAZIEGLER B, ZIEGLER A, TROWSDALE J AND TOWNSEND A. (1992). Assembly and function of the two ABC transporter proteins encoded in the human major histocompatibility complex. *Nature*, **355**, 641–644.

KOPP WC, SMITH JW, EWEL CH, ALVORD WG, MAIN C, GUYRE PM, STEIS RG, LONGO DL AND URBA WJ. (1993). Immunomodulatory effects of interferon-gamma in patients with metastatic malignant melanoma. *J. Immunother.*, **13**, 181–190.

LAGA M, ICENOGLE JP, MARSELLA R, MANOKA AT, NZILA N, RYDER, RW, VERMUND SH, HEYWARD WL, NELSON A AND REEVES WC. (1992). Genital papillomavirus infection and cervical dysplasia – opportunistic complications of HIV infection. *Int. J. Cancer*, **48**, 682–688.

LJUNGGREN H-G AND KARRE K. (1990). In search of the 'missing self':MHC molecules and NK recognition. *Immunol. Today*, **11**, 2327–244.

LJUNGGREN H-G, STAM NJ, OHLEN C, NEEFJES JJ, HOGLUND P, HEEMELS M-T, BASTIN J, SCHUMACHER TMN, TOWNSEND A, KARRE K AND PLOEGH HL. (1990). Empty MHC class I molecules come out in the cold. *Nature*, **346**, 476–480.

MELIEF CJM AND KAST WM. (1992). Lessons from T cell responses to virus induced tumours for cancer eradication in general. In *Cancer Surveys: A New Look at Tumour Immunology*, McMichael AJ and Bodmer WF. (eds) pp. 81–99. Cold Spring Harbor Laboratory Press: Cold Spring Harbor, NY.

MOLLER P AND HAMMERLING GJ. (1992). The role of HLA-A,B,C molecules in tumour immunity. *Cancer Surveys*, **13**, 101–127.

MONACO JJ. (1992). A molecular model of MHC class I-restricted antigen processing. *Immunol. Today*, **13**, 173–178.

MUNOZ N, BOSCH FX, SHAH KV AND MEHEUS A. (1992). *The Epidemiology of Cervical Cancer and Human Papillomavirus.* IARC Scientific Publications: Lyon.

NIEDOBITEK G AND HERBST H. (1991). Human papillomaviruses and cancer. *Cancer J.*, **4**, 163 167.

PHILLIPS RE, ROWLAND-JONES S, NIXON DF, GOTCH FM, EDWARDS JP, OGUNLESI AO, ELVIN JG, ROTHBARD JA, BANGHAM CRM, RIZZA CR AND McMICHAEL AJ. (1991). Human immunodeficiency virus genetic variation that can escape cytotoxic T cell recognition. *Nature*, **354**, 453–459.

SCHMIDT H, GEKELER V, HAAS H, ENGLER BLUM G, STEIRT I, PROBST H AND MULLER G. (1990). Differential regulation of HLA class I genes by interferon. *Immunogenetics*, **31**, 245–252.

SCHNIEDER A AND KOUTSKY L. (1992). Natural history and epidemiological features of genital HPV infection. In *The Epidemiology of Human Papillomavirus and Cervical Cancer*, Munoz N, Bosch FX, Shah KV and Meheus A. (eds) pp. 23–52. International Agency for Research on Cancer: Lyon.

SEUNG S, URBAN JL AND SCHREIBER H. (1993). A tumor escape variant that has lost one major histocompatibility complex class I restriction element induces specific CD8 + T cells to an antigen that no longer serves as a target. *J. Exp. Med.*, **178**, 933–940.

SMOTKIN D AND WETTSTEIN FO. (1987). The major human papillomavirus protein in cervical cancers is a cytoplasmic phosphoprotein. *J. Virol.*, **61**, 1351–1359.

STANLEY MA. (1994). Virus–keratinocyte interactions in the infectious cycle. In *Human Papillomaviruses and Cervical Cancer*, Stern PL and Stanley MA. (eds) pp. 116 131. Oxford University Press: Oxford.

STAUSS HJ AND BEVERLEY PCL. (1994). The search for cell-mediated immunity to HPV: prospect for vaccine design. In *Human Papillomaviruses and Cervical Cancer: Biology and Immunology*, Stern PL and Stanley MA. (eds). pp. 146–161. Oxford University Press: Oxford.

STERN PL AND DUGGAN-KEEN M. (1994). MHC expression in the natural history of cervical cancer. In *Human Papillomaviruses and Cervical Cancer. Biology and Immunology*, Stern PL and Stanley MA. (eds) pp. 162 176. Oxford University Press: Oxford.

TOWNSEND A, OHLEN C, BASTIN J, LJUNGGREN HG, FOSTER L AND KARRE K. (1989). Association of class I major histocompatibility and light chains indicated by viral peptides. *Nature*, **340**, 443–448.

TOWNSEND A, ELLIOTT T, CERUNDOLO V, FOSTER L, BARBER B AND TSE A. (1990). Assembly of MHC class I molecules analyzed *in vitro*. *Cell*, **62**, 285–295.

TROWSDALE J, HANSON I, MOCKRIDGE I, BECK S, TOWNSEND A AND KELLY A. (1990). Sequences encoded in the class II region of the MHC related to the ABC superfamily of transporters. *Nature*, **348**, 741–744.

VAN DEN BRULE AJC, MEIJER CJLM, BAKELS V, KENEMANS P AND WALBOOMERS JMM. (1990). Rapid human papillomavirus detection in cervical scrapes by combined general primers mediated and type specific polymerase chain reaction. *J. Clin. Microbiol.*, **28**, 2739–2743.

WEI ML AND CRESSWELL P. (1992). HLA-A2 molecules in an antigen-processing mutant cell contain signal sequence-derived peptides. *Nature*, **356**, 443 446.